# Positive behavior support in school – a quasi-experimental mixed methods study and a randomized controlled trial

**DOI:** 10.1186/s40359-024-02021-z

**Published:** 2024-10-01

**Authors:** Martin Karlberg, Nina Klang, Johanna Svahn

**Affiliations:** Department of Education, Uppsala university, Box 2136, Uppsala, 750 02 Sweden

**Keywords:** Quasi-experimental design, School-wide positive behavioral interventions and supports, SW-PBIS, School Climate, Intervention

## Abstract

**Background:**

While positive school climate is important for students’ well-being and mental health, school personnel may experience challenges in creating a nurturing school climate. School-Wide Positive Behavioral Interventions and Supports (SW-PBIS) has shown positive effects on school climate and children’s prosocial behaviors, but fewer studies have been conducted in a European context.

**Aim:**

This project aims to investigate the effectiveness of SW-PBIS program for students’ social-emotional skills and academic achievement as well as teachers’ and students’ perceptions of classroom learning environment. Furthermore, the study intends to evaluate how school-level factors mediate or moderate the effects of the intervention. In addition, the study includes a qualitative evaluation of the dynamic interaction processes that occur during program implementation in local school contexts.

**Methods:**

Data on school- and individual-level measures are collected in intervention and control schools. With regard to challenges in retaining control groups over extended time periods, two waves of recruitment are used. In the first wave, an active control group is used and data are collected during three time points. In the second wave, a wait-list control group will be used and data will be collected during two time points during one school year. Hierarchical regression analyses will be conducted to explore the effects of SW-PBIS on the outcomes of the study. An ethno-methodological approach will be applied to provide a detailed examination of the social interactional and meaning-making practices of different school implementation teams, and the negotiation of normative expectations and rules of conduct in peer-teacher-student interactions in different classrooms.

**Discussion:**

The study is expected to contribute to knowledge on the effects of the SW-PBIS program and how these effects may be mediated or moderated by school-level factors. Combining quantitative and qualitative methods to explore the significance of school contexts in the implementation of the SW-PBIS program constitutes the strength of the study. The challenge in the study is the extended period of implementation of SW-PBIS, which entails difficulties in retaining a control group over the required time period. Therefore, two waves of recruitment are used, encompassing different procedures of allocation to intervention or control group.

**Trial registration:**

ClinicalTrials.gov Identifier: NCT06270914 on the 22nd of February, 2024 (retrospectively registered).

**Supplementary Information:**

The online version contains supplementary material available at 10.1186/s40359-024-02021-z.

## Background

Creating a nurturing school and classroom climate is essential for students’ mental health, well-being and school achievement [1]. Poor classroom and school climate has a negative impact on students’ behaviors [e.g., 2, 3], while positive relationships with teachers, characterized by warmth and emotional support, may alter the course of negative development for children at risk [4, 5]. Furthermore, children who do not fully master the skills necessary to focus on school tasks and to get along with peers, may exhibit behaviors perceived as challenging. Schools’ response to these behaviors often involves disciplinary measures, leading to less instructional time necessary to achieve academically [e.g., 6]. In combination with a lack of nurturing relationships at school or at home this in turn puts this group of children at a heightened risk for school dropout, poor mental health and in the long run unemployment and for some even contacts with criminal justice [e.g., 7]. Thus, addressing school climate is urgent to prevent problem behaviors and promote positive school climate and mental health outcomes for children and youth.

The study focuses on the program School-Wide Positive Behavioral Interventions and Supports (SW-PBIS), which is a systems-level approach to address behavior problems and promote social behaviors by targeting school and classroom organization [8]. The program is grounded in multiple theories, including applied behavior analysis [9], social learning theory [10], coercion theory [11] and ecological theory [12]. The strength of the program is its collaborative nature, as it focuses on whole school prevention of behavior problems.

The evidence base of the SW-PBIS is well established. According to a recent review [13], the program resulted in reduction of behavior problems and increase in prosocial behaviors; as well as improvements in teachers’ and students’ perceptions of school climate. While research base for SW-PBIS in North American context is extensive, fewer studies have been published in Europe [14–16]. These studies have shown promising results concerning student and teacher behaviors, classroom climate, and peer relations. A study of SW-PBIS in Swedish schools reported no positive effects of the program [17]. The authors reported challenges in implementation as possible explanations for null results. Thus, it is yet left to be found out if, when and how SW-PBIS program is effective in Swedish school settings.

Previous studies also raise questions of the conditions under which the SW-PBIS program is effective, and for whom. First, at the individual level, the SW-PBIS program demonstrates higher effects for students who are at risk for behavior problems at baseline [14, 18]. Second, schools who implement the SW-PBIS with fidelity also appear to rip benefits of the program to a higher degree [13, 19]. School contextual factors (e.g., school size, location, team functioning) and teacher level factors (e.g., grade taught, perceived classroom climate) are associated with the variability in fidelity of implementation of SW-PBIS [20]. In addition, teacher self-efficacy has been outlined as one of the positive outcomes of SW-PBIS [21], but no study explored whether changes in teacher self- efficacy are related to changes in student behaviors. Thus, a consideration of multiple factors, would contribute to our understanding of how, when and for whom the SW-PBIS program is effective.

Social issues related to power asymmetries between teachers and students and ethical dilemmas are also crucial dimensions in social processes of implementation. These issues are something that further strengthens the need for a more detailed inquiry into how the core components in the SW-PBIS programs, as rules and behavioral expectations, are handled and negotiated in student-teacher interactions in the classroom. The complexity of teachers’ everyday classroom encounters has been brought about in studies of teacher-student interaction and co-construction of classroom norms from the perspectives of teachers and students [22, 23]. It is also important to focuse on teachers’ normative expectations in managing students’ challenging behavior and the emotionally charged nature of issues in classroom management [24]. The proposed project makes an important contribution to the field by an in-depth exploration of the processes that emerge and take form as teachers and students participate in the implementation of the SW-PBIS program.

## Aim

The aim of the project is twofold. Firstly, the aim is to evaluate the effectiveness of SWPBIS program for students’ social-emotional skills and academic achievement as well teachers’ and students’ perceptions of learning environment and to explore how school-level factors may moderate or mediate the effect of SWPBIS on the outcomes. Secondly, the aim is to get a deeper understanding of the social processes and local meanings that emerge and take form among the students, teachers and other school professionals as they participate in the SW-PBIS program. The aims are specified in four research questions.

- What are the effects of SW-PBIS program on students’ social-emotional skills and academic achievement as well teachers’ and students’ perceptions of learning environment?
- To what extent do school level factors mediate and moderate the effects of the SW-PBIS program on the study outcomes over time?
- How are the central contents of the SW-PBIS program made sense of and negotiated in school teams and classrooms at two schools during the course of program implementation?
- How do specific instances of student-teacher interaction lead to changes in students’ behavior and responses to the SW-PBIS program, and what forms of ethical dilemmas evolve in the management of behavior problems in classrooms?

## Methods: participants, interventions and outcomes

**Fig. 1:**
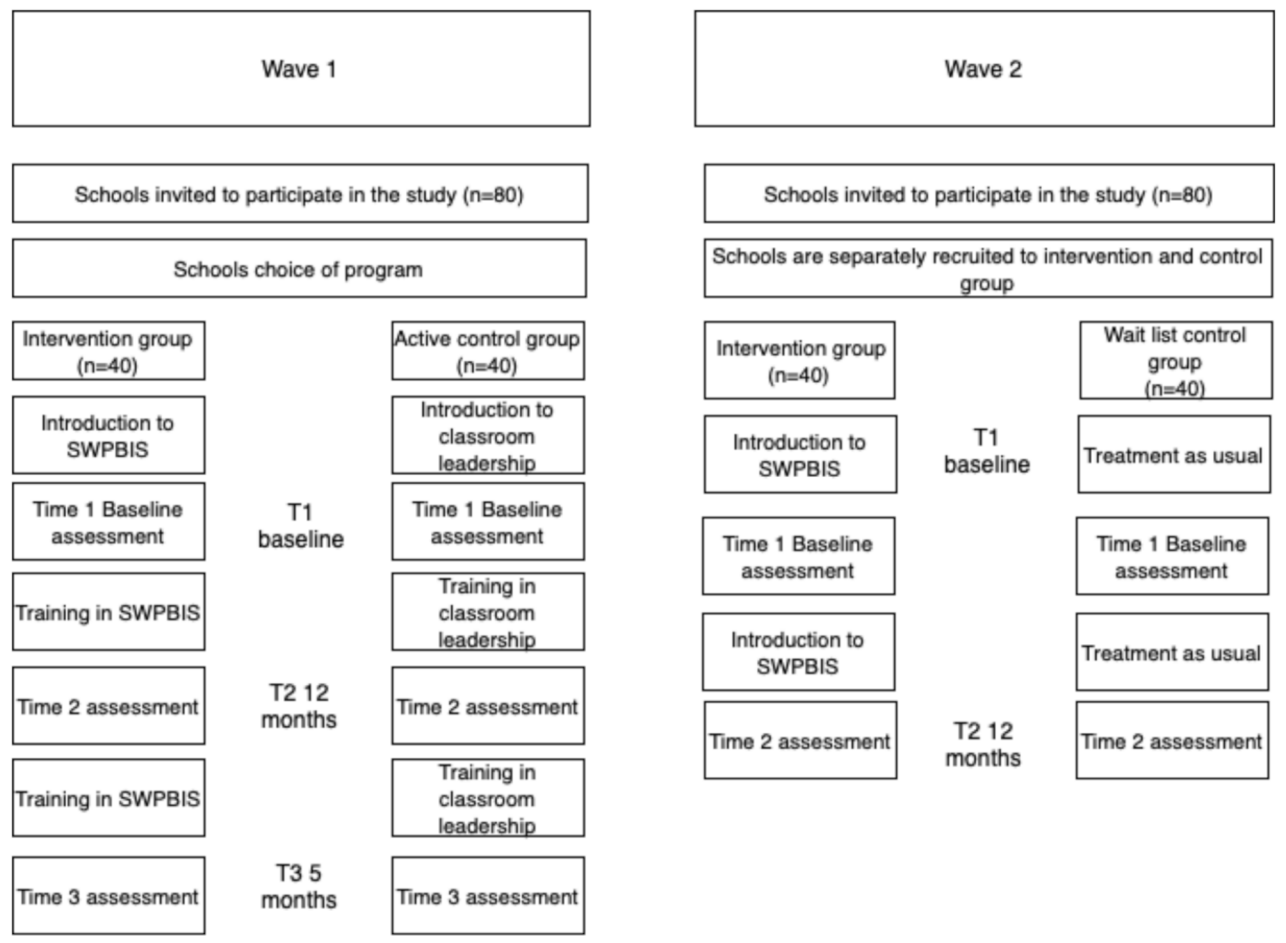
Two waves of data collection in the project (produced through the web-based application draw.io at www.diagrams.net (accessed on 30 May 2024)

This study includes two waves of participant recruitment with two designs: quasi-experimental non-randomized design (wave 1) and cluster-randomized design (wave 2). The intervention study has been approved by the Swedish Ethical Review Authority (2022-03482-01), and registered as a clinical trial at www.clinicalTrials.gov (identifier NCT06270914). The choice of two waves of recruitment is motivated by the challenge to retain a control group over two years, as previous studies have implemented SW-PBIS for three to four years [16, 18]. Therefore, two waves of recruitment are used. In the first wave of recruitment, intervention and active control group are used and outcomes will be measured at three time points. In addition to quantitative data, qualitative observations and interviews are conducted to get a deeper understanding of the process of implementation of SW-PBIS. In the second wave of data recruitment, intervention and wait-list control group are used and outcomes will be measured at two time points (pre- and post). In Fig. 1, recruitment and data collection for both waves of data collection are described.

### Participants

The study will be conducted in compulsory schools and include classes from grade one to grade nine. Inclusion criteria for the intervention and control groups are: (a) compulsory school, grades 1–9; (b) the school have not been previously enrolled in an SW-PBIS program by other providers (b) principal, teachers, students and their parents consent to participate in the study. Each of the two waves of recruitment in the study will include 80 schools. According to estimations with the software Optimal Design [25], 62 schools are required, based on an effect size of 0.25 and a power of 80%, with an expected amount of 12 classes per school, 20 students per class and an intraclass correlation (ICC) of 0.10 at school level and 0.05 at classroom level [26]. To account for 20% attrition, 80 schools are needed. Based on previous studies of SW-PBIS in the European context [14, 32], we expect Cohen’s d effect size of 0.20 to measure an important difference between the intervention and control group.

### Recruitment

Information about the study will be distributed by e-mail to all compulsory schools in Sweden. The e-mail lists will be retrieved from Swedish National Agency for Education. In addition, information about the study will be distributed at conferences and via regional principal and teacher networks. School principals and teachers will be invited to online meetings at which detailed information about the study will be presented and detailed information on participation in the study will be provided. Upon enrollment in the project, the information and invitation to participate in the study is distributed to students’ guardians. In addition to Swedish language, information about the study will be presented in ten frequent languages in Sweden (Arabic, Spanish, English, Somali, Serbo-Kroatian, Polish, Kurdish, Finnish, Farsi, and Urdu). Students in grade 9 will also be asked for informed consent, upon consent from both guardians.

In the first wave of recruitment, the decision to allocate participants to intervention or control group will be based on the school principals’ choice of intervention. The schools, catering for students in grades 4–9, which have expressed interest in the intervention, will be stratified in three groups, based on size, parent educational level, proportion of students with migrant background and students’ average achievement rates. This information is publicly available from SALSA database of the National Agency for Education. After stratification, schools within each stratum will be assigned to intervention group. The same procedure will be conducted in the recruitment of control groups. In the second wave of recruitment, the participants from schools grades 1–9 will be recruited the study, and then randomly allocated to either intervention or control group.

According to estimations with the software Optimal Design (OD; Spybrook et al., 2011), 62 schools are required, based on an effect size of 0.25 and a power of 80% with an expected amount of 12 classes per school, 20 students per class and an intraclass correlation (ICC) of 0.10 at school level and 0.05 at classroom level (Hedges & Hedberg, 2007). To account for 20% attrition, 74 schools are needed (approximately 17 280 students). Therefore, in each wave of data collection, a minimum of 74 schools will be recruited.

### Intervention group

The intervention is based on the Norwegian manualized program of SW-PBIS, N-PALS (Positiv Atferd, Støttende Læringsmiljø, og Sammhandling). The Norwegian model shares its core components with the original SW-PBIS model [16]. The program’s core components constitute: (a) positive behavior support strategies, including positive expectations and classroom rules, which are followed up with positive feedback and encouragement; (b) a system for monitoring student behavior, (c) school-wide corrections using consequences, (c) instruction in classroom management skills for teachers, and (e) strategies for collaboration with parents. These components are implemented through activities at school level and at classroom level. The implementation at school level starts with establishing a leadership team, comprised of school leaders, team leaders and members of school welfare team. The leadership team assesses the needs and resources at the school and agrees on behavioral expectations and rules. The team monitors the student behaviors and provides opportunities for teacher professional development with focus on classroom management and relationship building. At classroom level, the teachers incorporate school-wide expected behaviors into their classroom instruction through classroom activities and classroom rules. The teachers also integrate their training in classroom management and relationship building in their teaching practices.

The training in the project is primarily provided through instructors, that are appointed at each school. The trainers attend online training sessions for ten afternoons over eight months. Depending on the size of the school, the schools in the SW-PBIS group send between two and ten staff members for SW-PBIS instructor training (ten training sessions, totalling 25 h). The instructors then educate and supervise the school staff, with the support of the SW-PBIS team. The instructor is in turn responsible for training and mentoring school staff in SW-PBIS. Instructors are supported in the implementation of a SW-PBIS team in the school. The team includes the school’s principal, student health staff and representatives of the school’s teams and professions. The team has two important tasks: to participate in the planning of the implementation and facilitate the work of the instructors, and to contribute to the adaptation of the program to local conditions. Instructors teach and mentor school staff 1–2 times a month (approximately 1.5 h per session) and meet regularly with the SW-PBIS team. The manual for the universal Tier 1 of the Norwegian N-PALS program was translated into Swedish and adapted to Swedish conditions [27]. The program was first pilot-tested in nine schools [28]. Prior to the implementation of the program, three instructors and two researchers (Martin Karlberg and Nina Klang) received training at the Norwegian Center for Child Behavioral Development.

### Control group

The control group in the first wave of data collection is selected in accordance with quasi-experimental design with a nonequivalent control group [29]. The teachers in the control group will, instead of a wait-list intervention, receive a shortened version of SW-PBIS, containing only classroom management. The schools that are part of the classroom management group also send staff for instructor training (ten training sessions, 25 h in total, just as much as the SW-PBIS schools). Similar to the SW-PBIS group, the instructors in this group educate and supervise school staff, supported by a team at the school. Both groups have access to a manual and supporting materials. This design does not pose threats to the internal validity of the study, as classroom management is one of principles of PBIS framework. In this way it will be possible to explore the specific contribution of SW-PBIS in contrast to classroom managment only. Control group in the second wave of data collection constitutes a wait-list control group and will not be engaged in any kind of intervention during the intervention period.

### Outcome measures

Outcome measures encompass both individual-level and school-level measures. Individual-level measures constitute the primary outcome of the intervention, as the intention in the study is to evaluate whether the intervention benefits the participating students, while school-level variables are primarily used to explain the potential effect of the intervention. All the outcomes are assessed by researchers using a survey methodology. See Table 1 for a list of all measurement points.

**Table 1:** Data on outcome measures at both individual and school level will be used

	2023	2024	2025
**Wave 1 Recruitment**			
Social skills improvement system – SEL Brief and Mental Health Scales – Teacher Form	x	x	x
Learning environment – Teacher and Student Questionnaire	x	x	x
Students’ achievement in national tests	x		x
Teacher Collective Self-efficacy	x	x	x
Problem behavior in classroom and school	x	x	x
**Wave 2 Recruitment**			
Social skills improvement system – SEL Brief and Mental Health Scales – Teacher Form		x	x
Learning environment – Teacher and Student Questionnaire		x	x
Teacher Collective Self-efficacy		x	x
Problem behavior in classroom and school		x	x

### Individual level measures

At individual level measures constitute teacher ratings of students’ social-emotional skills, behavior problems and academic competence, using Social Skills Improvement System – SEL Brief and Mental Health Scales, Teacher K-12 Form [33] as well as teacher and student questionnaires of classroom learning climate [32, 34].

**Social emotional skills.** Social Skills Improvement System – SEL Brief and Mental Health Scales, Teacher K-12 Form [33] contains 20 items related to five competency domains (self-awareness, self-management, social awareness, relationship skills, and responsible decision making) and 10 items encompassing emotional behavior concerns (internalizing and externalizing concerns). Examples of items are “Asks for help when needed” for social-emotional skills competency domains and “Withdraws from others” for emotional behavior concerns. The items are measured on a 4-point scale (1 = Never, 2 = Seldom, 3 = Often, 4 = Almost always). The measures have proved to have sufficient validity and reliability in previous studies [35, 36].

**Classroom learning environment.** A comprehensive assessment of the classroom learning environment is conducted using questionnaires for teachers and students [32, 34]. The teacher questionnaire consists of 14 statements, rated on a 4-point scale (1 = does not fit to 4 = fits completely), assessing various aspects of the classroom learning climate. The student questionnaire includes 22 statements, also rated on a 4-point scale, gauging students’ perceptions of the classroom environment. An example of an item in the teacher questionnaire is “There is a good working climate during lessons”. An example of an item in the student questionnaire is “Students in this class are active and interested during lessons”.

**Students’ achievement.** In addition, for the first wave of recruitment, students’ achievement on national tests of Swedish and mathematics will be retrieved from register data provided by Statistics Sweden. The data will be standardized across tests and compared with regard to the national average for those students who enter the project in grade 4 and 7 (national tests in grade 3 and grade 6) and finish the project in grade 6 and 9 (national tests in grade 6 and grade 9).

### School level measures

At school level measures encompass Teacher Collective Self-efficacy [30], and school level measures of problem behavior [31, 32].

**Collective self-efficacy.** The Collective Self-efficacy questionnaire (CES; 30) will be used as a measure of teacher collective beliefs of possibilities to influence student learning. CES contains 21 items and a 6-point scale is used (from 1 = Do not agree at all, 2 = Do not agree, 3 = Do not agree fully, 4 = Agree a little, 5 = Agree, 6 = Agree completely). An example of item is “Teachers in this school are able to get through to difficult students”. In previous studies, collective self-efficacy has shown to be influenced by SW-PBIS programs [16].

**Problem behavior in classroom and school.** The Problem Behavior in the Classroom Last Week scale (20 items) is used to collect reports of disruptive behavior in the classrooms on a 5-point Likert scale from 0 (not observed) to 5 (observed several times per day) [31, 32]. The Problem Behavior in the School Environment scale [31, 32] is a school level measure containing 15 items and similar rating alternatives. Examples of behaviors in the scales are “Destroying school environment” or “Physical aggression towards peers and teachers”. The scales have shown satisfactory psychometric properties in prior studies in Norway [e.g., 32].

### Implementation fidelity

To measure implementation fidelity of the SW-PBIS Tier 1, the members of school leadership team will be asked to complete questionnaire Benchmarks of Quality [37] each year. The questionnaire is a self-evaluation tool that supports school professionals in monitoring progress of implementation of SW-PBIS Tier 1. BoQ contains 53 items, divided into 10 subscales covering team functioning, staff commitment, expectations and discipline procedures. Each item can be rated on a 3-point scale (1 = Not yet, 2 = Can be better, 3 = Is in place). The items are summarized in a total score.

### Data analyses

Hierarchical regression analyses with a three-level structure [38] will be conducted to explore the effects of the SW-PBIS on study outcomes. Hierarchical regression analyses allow to account for the nested structure of the data, in which teachers’ and students’ responses are nested within schools and grade levels. The outcomes at school and individual level will be studied as a function of time (three time points for wave 1 and two time points for wave 2) and group belonging (intervention or control group). To take into account the nestedness of the data, three-level random effect model will be used, varying by school, grade and the individuals’ identification codes. Furthermore, the possible mediators (quality of implementation) and moderators (school characteristics, school-level variables) of the SW-PBIS intervention will be analyzed. Missing data will be handled by imputation, provided that the assumption of data missing completely at random is met.

### Qualitative evaluation

In the qualitative study, we use a longitudinal ethnographic approach in order to obtain detailed contextualized knowledge on interactions in various contexts in two of the intervention schools, recruited in wave 1, over a longer period of time. Through purposeful sampling, three schools with different characteristics regarding size and catchment area will be selected.

Ethnographic fieldwork will be conducted in two of the intervention schools starting from the sixth month of SW-PBS introduction. The ethnographic approach will target the ongoing work of the school implementation teams and will include participant observations and interviews. In addition, one class at each school will be selected for further observations, teacher and student interviews, and more detailed video-documentation of classroom interactions [39]. The two ethnographic field studies will proceed over a period of two years in order to enable detailed analysis of continuous social processes, consequences and results of the intervention program.

The qualitative analytic tools, based on, multimodal ethnomethodology and conversation analysis [e.g., 39, 40], enables us to obtain detailed contextualized knowledge on interactions between members of the school implementation teams as well as ongoing teacher-student interactions within the classrooms. Such an interactional approach is particularly fruitful for examining how specific interactional moments lead to changes in the participants’ responses to the SW-PBIS, and how the central elements of the intervention program, including preexisting expectations and common rules, are talked-into-being, but also negotiated and reconstructed in actual interactions. The use of longitudinal video data of classroom interactions will provide possibilities to investigate in what ways the intervention program affects the relationships between students and teachers, and leads to changes across time and space [41]. In the longitudinal analysis, specific focus will also be given to the ethical dilemmas and challenges that emerge in the teachers’ and students’ everyday interactions, induced by the implementation of the SW-PBIS program at the different schools.

### Ethics and dissemination

The study was granted Ethical approval by the Swedish Ethical Review Authority (2022-03482-01; 2023-02231-02; 2024-02383-02). Information about the study will be provided in accordance with the Swedish Ethical Review Authority, including voluntary participation, confidentiality, data storage and a nuanced description of risks of participating in the intervention. Informed consent will be collected from students’ guardians prior to participation in the study. Informed consent from principals and teachers will be collected in connection to surveys. To ensure confidentiality, students and teachers will be assigned individual codes and code keys will be stored separately from the collected data. The data will be deposited at a secure storage place at Uppsala University and will be restricted to the research team. A plan for documenting and addressing potential adverse events and other unexpected effects of the intervention is in place. In case of possible adverse events, the researchers give extended support to the schools (by an additional meeting) and if considered necessary, the school discontinues participation in the intervention. All adverse events or negative effects will be documented. In case of possible important protocol modifications, an application will be submitted to the National Ethical Authority. No data monitoring committee is appointed, as the researchers share responsibility for data monitoring (NK and MK for quantitative study, JS for qualitative study).

The results of the study will be published in scientific journals and practice-oriented magazines. The results will also be presented at national and international conferences.

## Discussion

The study aims to evaluate the effectiveness of SW-PBIS program for students’ social-emotional skills and academic achievement as well teachers’ and students’ perceptions of learning environment. Furthermore, the study intends to explore how school-level factors may moderate or mediate the effect of SW-PBIS on the outcomes. We hypothesize that the effects of SW-PBIS may vary dependent on school-level factors characterizing the schools participating in the project. The strength of the study further lies in the combination of quantitative and qualitative methods, which may enable us to understand possibilities and challenges for implementation of SW-PBIS program in varied school contexts and relate it to study outcomes. The challenge of the study lies in the fact that the implementation of SW-PBIS is extended over a longer period of time at the same time as it is difficult to retain control groups over such extended periods of time. Therefore, two waves of recruitment are used, in which active control group is retained over two years while wait-list control group is retained over a school year. The advantage in the first wave of recruitment is that it allows for a longer term evaluation, but the challenge is the lack of full experimental control. The advantage in the second wave of recruitment is that it allows for experimental control but is restricted to a short term evaluaton of the SW-PBIS model.

## Electronic supplementary material

Below is the link to the electronic supplementary material.

Supplementary Material 1: Overview of the study based on the SPIRIT Outcomes 2013 and 2022 checklist

## Data Availability

The datasets generated and/or analysed during the current study are not publicly available due to the protection of individual students and teachers participating in the study but are available from the corresponding author on reasonable request.

## Abbreviations

SW-PBIS: School-Wide Positive Behavior and Intervention Supports
